# High expression of TCN1 is a negative prognostic biomarker and can predict neoadjuvant chemosensitivity of colon cancer

**DOI:** 10.1038/s41598-020-68150-8

**Published:** 2020-07-20

**Authors:** Guang-jie Liu, Yao-jie Wang, Meng Yue, Lian-mei Zhao, Yong-Dong Guo, Yue-ping Liu, Hui-chai Yang, Fang Liu, Xue Zhang, Liang-hui Zhi, Jing Zhao, Yan-Hua Sun, Gui-ying Wang

**Affiliations:** 1 Department of Thoracic Surgery, Hebei Medical University Fourth Affiliated Hospital, Shijiazhuang, 050001 Hebei China; 2 Department of Research Center, Hebei Medical University Fourth Affiliated Hospital, Shijiazhuang, 050001 Hebei China; 3 Department of Pathology, Hebei Medical University Fourth Affiliated Hospital, Shijiazhuang, 050001 Hebei China; 4 Department of Medical Oncology, Hebei Medical University Fourth Affiliated Hospital, Shijiazhuang, 050001 Hebei China; 5 Department of General Surgery, 980th Hospital of Hoint Logistic Support Force, Shijiazhuang, 050001 Hebei China; 6 Department of Anorectal Surgery, Hebei Medical University Second Affiliated Hospital, Shijiazhuang, 050001 Hebei China; 7 Gastrointestinal Hernia Surgery, Cangzhou People’s Hospital, Cangzhou, 050001 Hebei China; 8 Department of The Second General Surgery, Hebei Medical University Fourth Affiliated Hospital, Shijiazhuang, 050001 Hebei China

**Keywords:** Cancer, Biomarkers, Oncology

## Abstract

Transcobalamin (TCN1) is a vitamin B12 (cobalamin)-binding protein that regulates cobalamin homeostasis. Recent studies and bioinformatic analyses have found that *TCN1* is highly expressed in cancer tissues and is associated with tumour aggressiveness and poor prognosis. The present study aimed to detect *TCN1* as a novel biomarker for prognosis and chemosensitivity of colon cancer. Next-generation sequencing showed that *TCN1* was one of several upregulated mRNAs in colon cancer, which was verified by further bioinformatics analyses. Western blotting (n = 9) and quantitative real time polymerase chain reaction (qRT-PCR, n = 30) revealed that *TCN1* was highly expressed in colon cancer tissues at both the protein and mRNA level. A total of 194 cases of colon cancer were examined by immunohistochemistry and revealed that TCN1 expression level was related to advanced stages (*P* < 0.005). Kaplan–Meier analysis verified that patients with lower TCN1 expression usually had longer overall survival (*P* = 0.008). In addition, TCN1 was highly expressed in pulmonary metastatic tumour tissues (n = 37, *P* = 0.025) and exhibited higher levels in right-sided colon cancer than in left-sided colon cancer (*P* = 0.029). TCN1 expression in specimens that had received neoadjuvant chemotherapy decreased compared with that in colonoscopy biopsy tissues (n = 42, *P* = 0.009). Further bioinformatics analyses verified that apoptosis pathways might have a role in high *TCN1* expression. All the studies revealed that *TCN1* expression in colon cancer was significantly associated with malignant biological behaviour. Therefore, TCN1 could be used as a novel biomarker for colon cancer aggressiveness and prognosis and might also be a potential biomarker for predicting neoadjuvant chemosensitivity.

## Introduction

According to the data from GLOBOCAN 2018, colorectal cancer (CRC) is one of the most common intestinal tumours and ranks as the fourth leading cause of morbidity and the second leading cause of cancer-related mortality worldwide^1^. Despite major developments in surgery and therapeutics, long-term survival remains far from satisfactory^2,3^, mainly because CRC is often detected at more advanced stages. Currently, diagnosis, relapse, and metastasis monitoring of CRC largely relies on colonoscopy and imaging data, which is usually delayed. Therefore, new sensitive biomarkers are urgently required to ensure early diagnosis and to predict progression and administer timely treatments.

Transcobalamin I (TCN1) is a type of vitamin B12 (cobalamin)-binding protein that transports cobalamin from the stomach to the intestines. It plays various roles in maintaining the basic function of cell proliferation and metabolism, especially in haematopoiesis and the nervous system^4,5^. Researchers have found that high levels of cobalamin and TCN1 in human serum are associated with leukaemia, hepatocellular carcinoma, and phyllodes of breast tumours ^6–8^. Overexpression of TCN1 in tumour tissues is associated with tumorigenesis and poor biological behaviour^9^. Chu et al. showed that *TCN1* is an oncogene, ranking as one of the top six differentially expressed mRNAs in CRC^10^. Feodorova et al. revealed that *NOTUM*, *TCN1*, *MACC1*, *YKL40*, *GPC3*, *AXIN2*, and *IL6* are significantly upregulated in CRC and have roles in tumour invasion and metastasis^11^. Our previous next-generation sequencing (NGS) results verified that *TCN1* is one of the top five overexpressed mRNAs in CRC^12^. Taken together, these findings indicate that *TCN1* is a CRC-related gene that requires further study. To our knowledge, the present study is the first to investigate the relationship between *TCN1* expression and the clinical behaviour of colon cancer.

NGS, western blotting, and immunohistochemistry (IHC) revealed that TCN1 was highly expressed in colon cancer tissues compared with adjacent normal mucosal tissues at both the protein and mRNA level. IHC staining of tissue microarray (TMA) slices also verified that TCN1 was highly expressed in colon cancer tissues (73.20%, 142/194) and pulmonary metastatic tumours (83.78%, 31/37). We also found that the expression of TCN1 was higher in right-sided colon cancer than in left-sided. High levels of TCN1 expression were associated with advanced pathological features and poor prognosis. Furthermore, TCN1 expression levels were decreased after neoadjuvant chemotherapy, which provided evidence that TCN1 might have a role in predicting chemosensitivity. Thus, we can conclude that high expression of TCN1 is significantly correlated with advanced clinicopathological features and poor prognosis of colon cancer. It may be novel biomarker for progression and prognosis and might be a potential biomarker to predict neoadjuvant chemosensitivity of colon cancer.

## Results

### *TCN1* is overexpressed in CRC tissues compared with adjacent mucosal tissues

**Figure 1:**
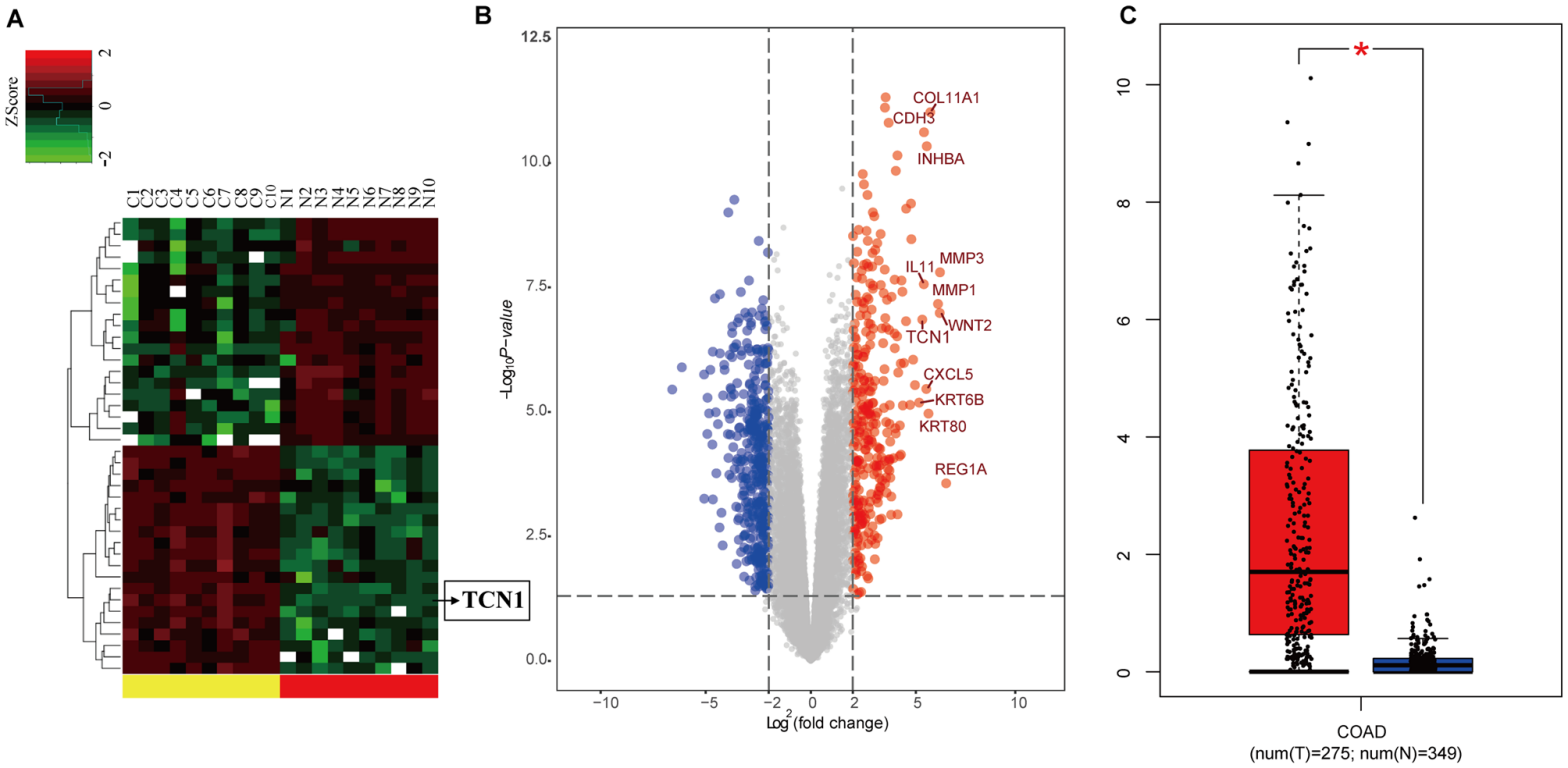
Next generation screening of *TCN1* mRNA expression in 10 cases of CRC and adjacent normal mucosal tissues and database verification. (**A**) Heat map of the top 20 differentially expressed mRNAs. (**B**) Volcano plot of differential mRNA expression (|log_2_FoldChange|> 2 and adjusted *P* < 0.05). (**C**) COAD data set verification of *TCN1* expression in CRC.

Differentially expressed mRNAs in CRC and paired adjacent normal mucosal tissues were screened by NGS (GSE104836)^12^. A total of 706 mRNAs (310 upregulated and 396 downregulated) were differentially expressed in CRC tissues (|log_2_FoldChange|> 2 and adjusted *P* < 0.05). A heat map and hierarchical clustering (|log_2_FoldChange|> 2 and adjusted *P* < 0.05) revealed the top 20 differentially expressed mRNAs (Fig. 1A), while a volcano plot (|log_2_FoldChange|> 2 and adjusted *P* < 0.05) indicated the differences in gene expression (Fig. 1B). The top five upregulated mRNAs were *REG1B*, *TCN1*, *MMP7*, *CST1*, and *SLCO1B3*, and the top five downregulated mRNAs were *ADIPOQ*, *OTOP2*, *KRT24*, *RERGL*, and *CA7*. *TCN1* was ranked the second highest upregulated mRNA. Based on the COAD cohort in the GEPIA database, further bioinformatics analyses were performed and confirmed that *TCN1* was significantly upregulated in CRC tissues (n = 275) compared with non-tumour tissues (n = 349) (*P* < 0.05, Fig. 1C).

### TCN1 was confirmed to be highly expressed in human colon cancer tissues

**Figure 2:**
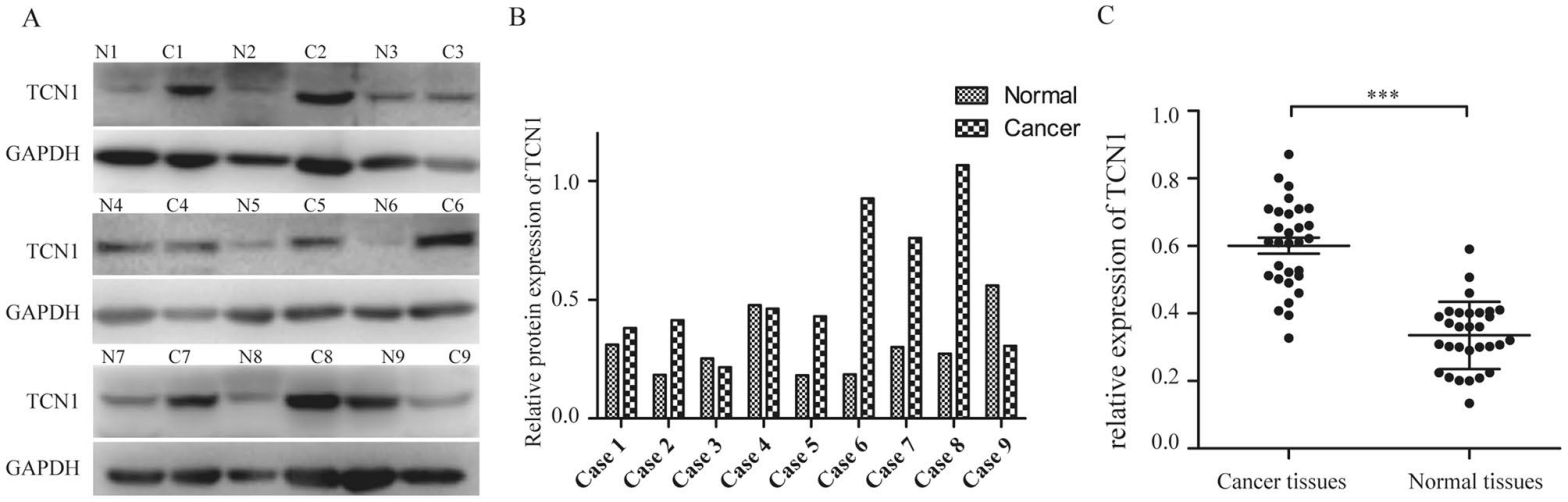
TCN1 expression in colon cancer. (**A**, **B**) Western blot analysis of TCN1 in 9 cases of colon cancer. (**C**) qRT-PCR analysis of *TCN1* mRNA in 30 cases of colon cancer versus normal tissues (***P < 0.001).

Western blot and qRT-PCR assays revealed that TCN1 was highly expressed in most of colon cancer tissues both at the protein (Fig. 2A, B) and mRNA level (Fig. 2C), but was poorly expressed in adjacent normal colon mucosal tissues. The relative mRNA expression level of *TCN1* in cancer tissues was higher than in non-cancerous tissues (0.601 ± 0.024 vs. 0.335 ± 0.018, *P* < 0.001).

### Increased TCN1 expression was associated with clinicopathological features and poor prognosis of colon cancer

**Figure 3:**
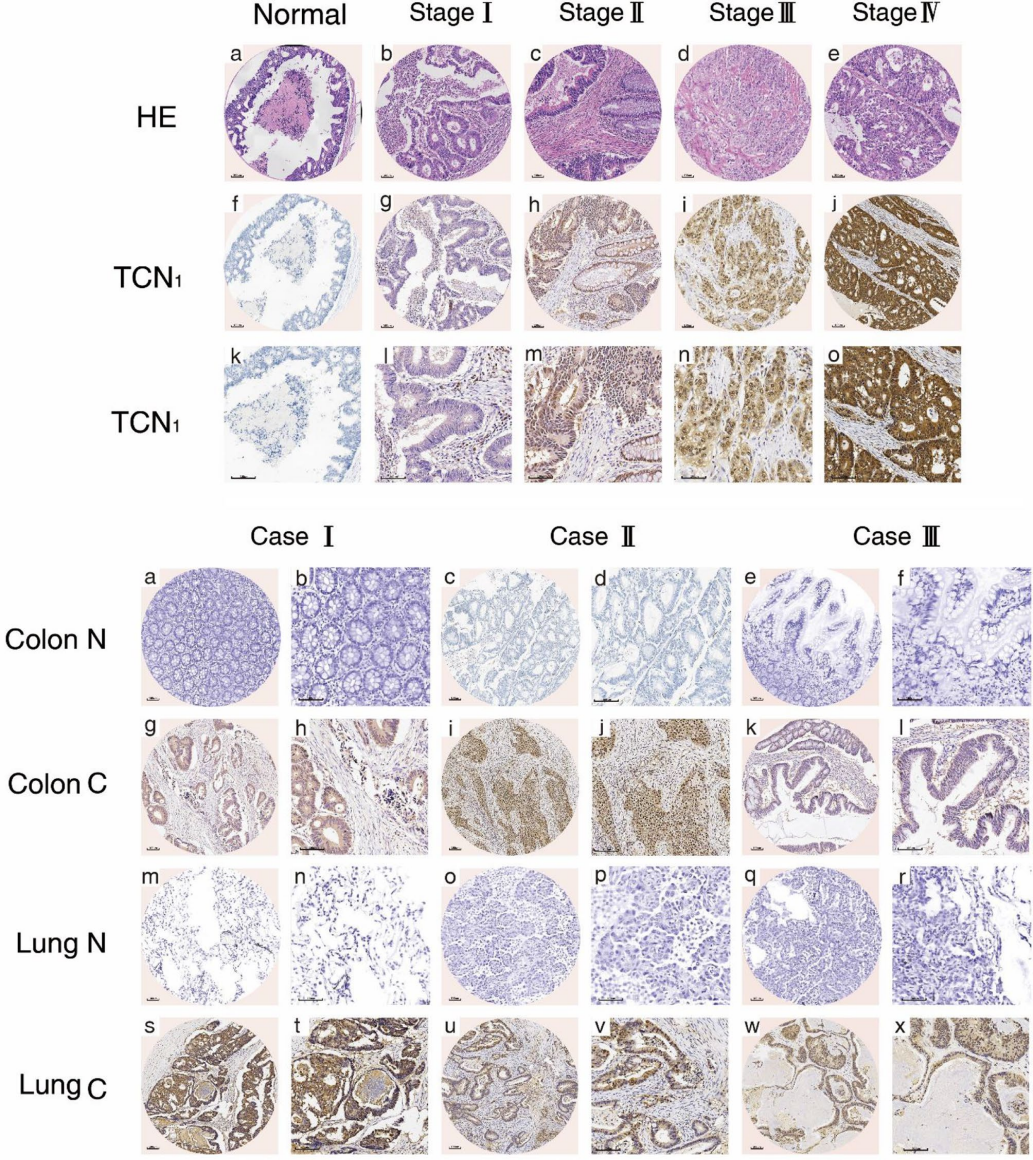
Representative IHC staining of different tissues. (**a**) Representative haematoxylin–eosin (HE) and IHC staining images for distinct stages and staining degree of colon cancer. (**b**) Representative IHC images of paired colon cancer, pulmonary metastatic, and adjacent normal tissues.

**Figure 4:**
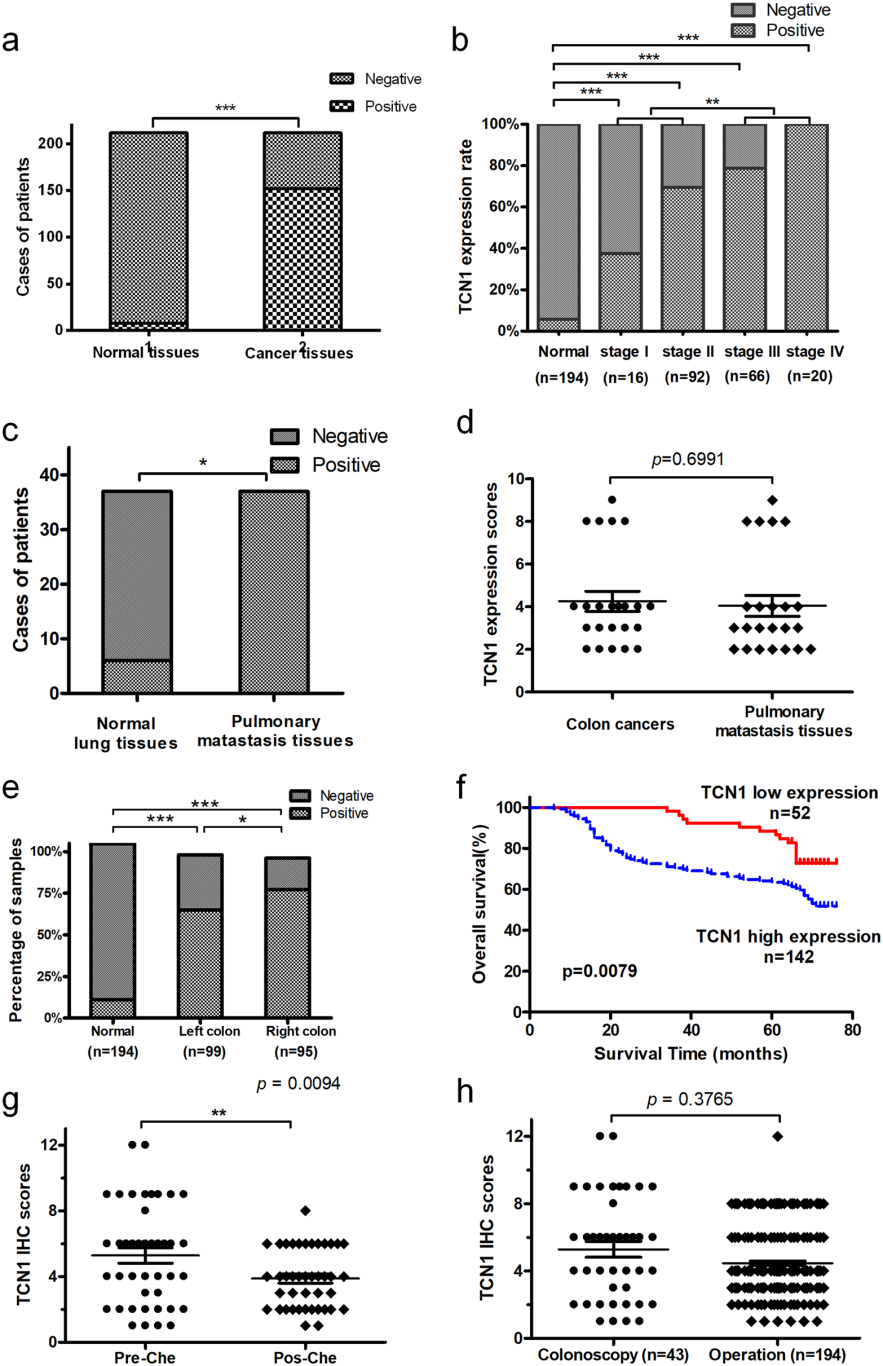
Increased TCN1 expression is associated with clinicopathological features, poor prognosis, and chemosensitivity of colon cancer. (**a**) TCN1 expression in colon cancer and adjacent normal mucosal tissues. (**b**) Expression status of distinct colon cancer clinical stage and adjacent normal mucosal tissues. (**c**) Expression of TCN1 in pulmonary metastatic tissues (PMT) and paired normal lung tissues (NLT). (**d**) Comparison of TCN1 expression in colon cancer and PMT. (**e**) Expression of TCN1 in left- and right-sided colon cancer. (**f**) Effect of TCN1 expression level on overall survival. (**g**) TCN1 expression level was decreased after platinum-containing chemotherapy (pre-chemical therapy vs post-chemical therapy). (**h**) TCN1 expression in colonoscopy biopsy tissues and specimens resected during surgery. *P < 0.05; **P < 0.01; ***P < 0.001.

The clinicopathological characteristics of the 194 colon cancer patients are presented in Table 1. IHC staining was performed on TMA slides, and immunoreactivity of TCN1 was observed primarily in the cytoplasm (Fig. 3). TCN1 expression was significantly higher in colon cancer tissues than in adjacent normal tissues (73.20% vs. 5.67%, *P* < 0.01, Fig. 4a). Furthermore, positive TCN1 expression was significantly correlated with T, N, M, TNM stage and age (Fig. 4b and Table1) and was higher in right-sided colon tumors than in left-sided tumors (Fig. 4e, Table 1). It was much higher in lung metastatic tissues (Fig. 4c) and the expression was consistent with that of the primer colon cancer tissues (Fig. 4d). Furthermore, Kaplan–Meier survival analysis demonstrated that higher TCN1 expression in patients represented significantly poorer OS (Fig. 4f). Univariate analysis showed that age, TNM staging and TCN1 expression were prognostic factors, while multivariate prognostic analysis showed that TCN1 expression level and M staging were independent prognostic factors (Table 2).

**Table 1:** Relationship between TCN1 expression and clinicopathological parameters of 194 colonic cancer patients.

Parameter	TCN1 expression (n)		χ^2^ value	*P* value
	Negative	Positive		
**Gender**				
Male	25	72	0.105	0.746
Female	27	70		
**Age**				
≤ 65	27	100	5.761	0.016
> 65	25	42		
**Tumor location**				
Left sided	33	65	4.763	0.029
Right sided	19	77		
**T classification**				
T1–T2	9	7	7.707	0.006
T3–T4	43	135		
**N classification**				
N0	38	72	7.760	0.005
N1–N2	14	70		
**M classification**				
M0	52	121	8.624	0.003
M1	0	21		
**TNM stage**				
I–II	38	70	8.722	0.003
III–IV	14	72		
**Drinking history**				
Yes	14	39	0.006	0.940
No	38	103		
**Smoking history**				
Yes	13	36	0.002	0.960
No	39	106		
**Pathological type**				
Adenocarcinoma	40	114	0.262	0.609
Mucinous adenocarcinoma	12	28		
**Lung metastatic and normal tissues**				
Lung metastatic tissues	0	37	4.811	0.025
Lung normal tissues	6	31

**Table 2:** Univariate and multivariate analysis of different prognostic factors for overall survival of 194 colonic cancer patients.

	Univariate analysis			Multivariate analysis		
	HR	95% CI	*P* value	HR	95% CI	*P* value
Gender	0.594	0.611–1.487	0.834			
Age	0.969	0.608–1.545	0.895	–	–	–
Cancer location	0.800	0.513–1.247	0.324	–	–	–
Pathology type	1.202	0.674–2.142	0.534			
Smoking history	0.923	0.550–1.548	0.762	–	–	–
Drinking history	0.860	0.517–1.430	0.561	–	–	–
T classification	1.179	0.513–2.712	0.699	–	–	–
N classification	1.725	1.105–2.692	0.016	2.155	0.847–5.479	0.107
TNM Stage	1.569	1.006–2.447	0.047	0.599	0.227–1.583	0.301
M classification	2.456	1.351–4.466	0.003	2.020	1.030–3.963	0.041
Expression states	2.189	1.206–3.973	0.010	1.855	1.003–3.432	0.047

### TCN1 expression level decreases after chemotherapy

After treatment with platinum-containing neoadjuvant chemotherapy, TCN1 expression in tumour tissues was decreased(*P* = 0.009, Fig. 4g). There was no significant difference in TCN1 expression status between colonoscopy biopsy tissues and specimens removed during surgery (*P* = 0.3765, Fig. 4h).

### GO (Gene Ontology) and Kyoto Encyclopedia of Genes and Genomes (KEGG) analysis results of *TCN1*-related genes in CRC

**Figure 5:**
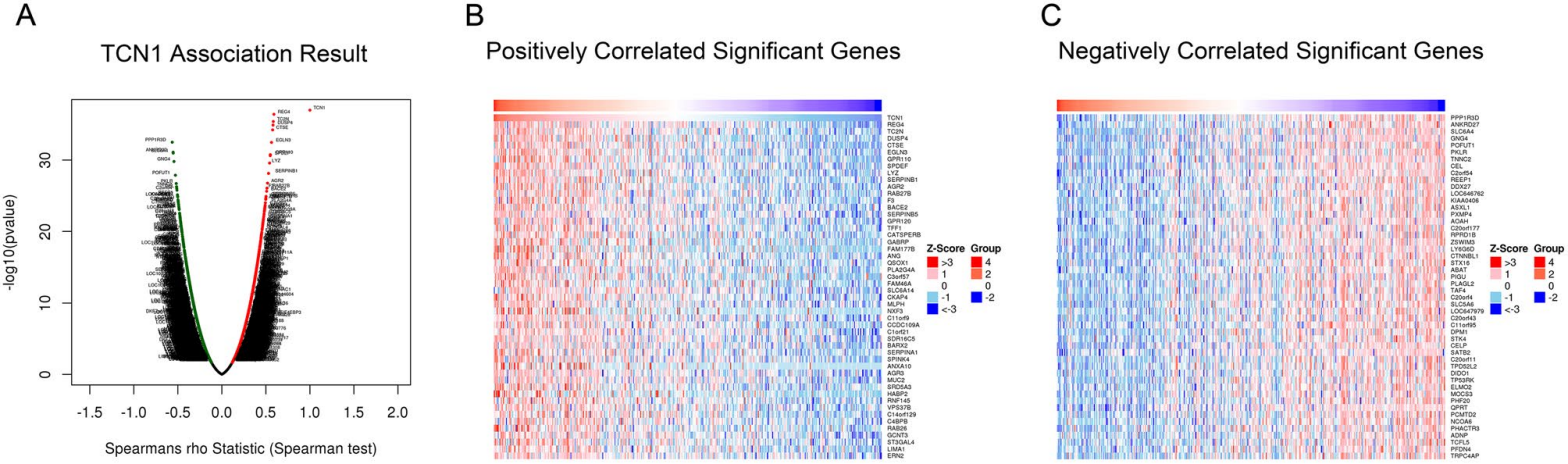
Differentially expressed genes correlated with *TCN1* in CRC based on the LinkedOmics database. (**A**) Hierarchical clustering is presented to show the differentially expressed genes correlated with *TCN1*. (**B**, **C**) The top 50 genes positively and negatively correlated with *TCN1* are presented in heat maps. Red and blue represent genes positively and negatively correlated with *TCN1*, respectively.

**Figure 6:**
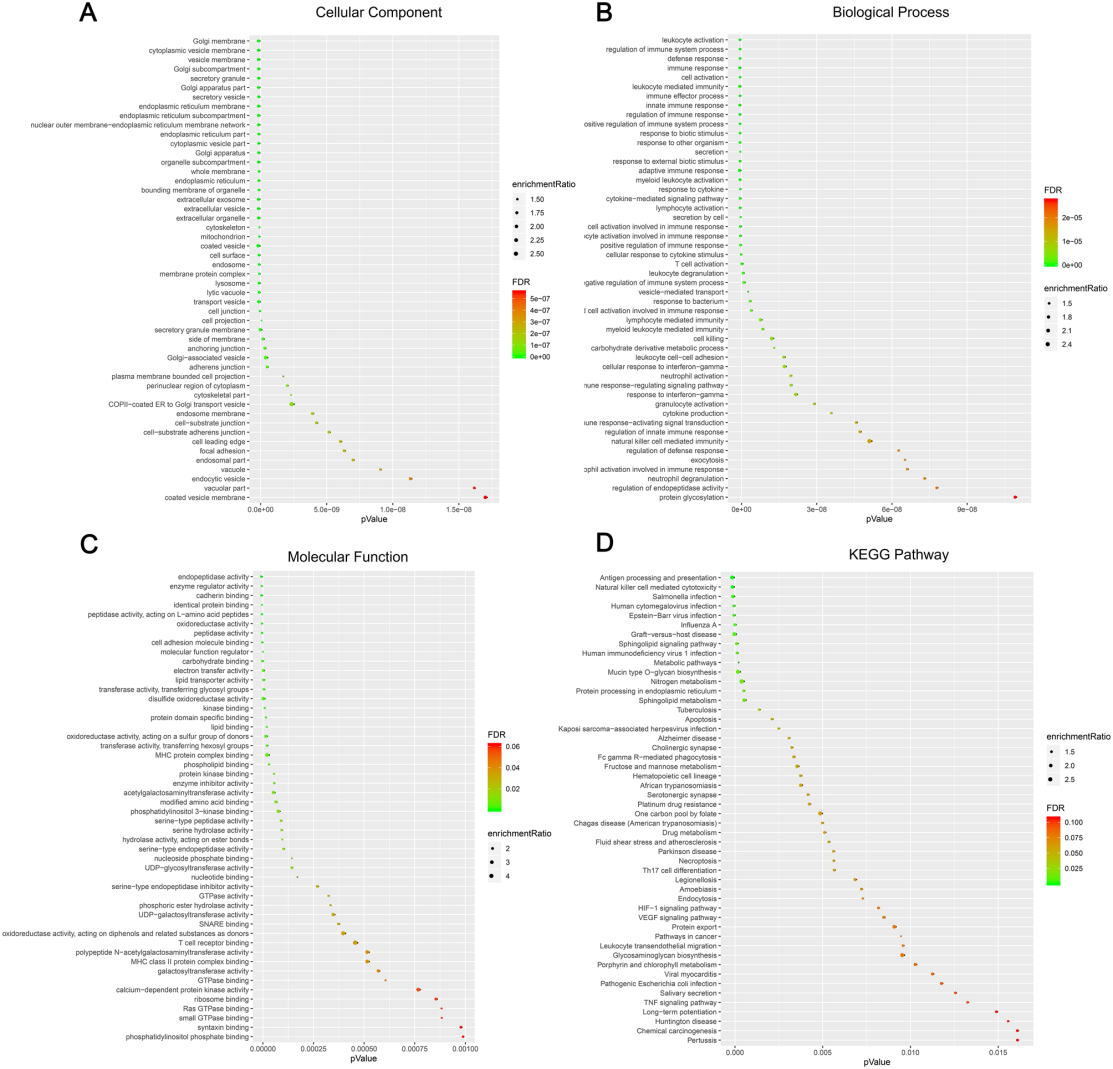
Genes positively correlated with *TCN1. *Genes positively correlated with *TCN1* as represented by over-representation analysis (ORA) and classified by (**A**) cellular components, (**B**) biological processes, (**C**) molecular functions, and (**D**) KEGG pathway analysis.

**Figure 7:**
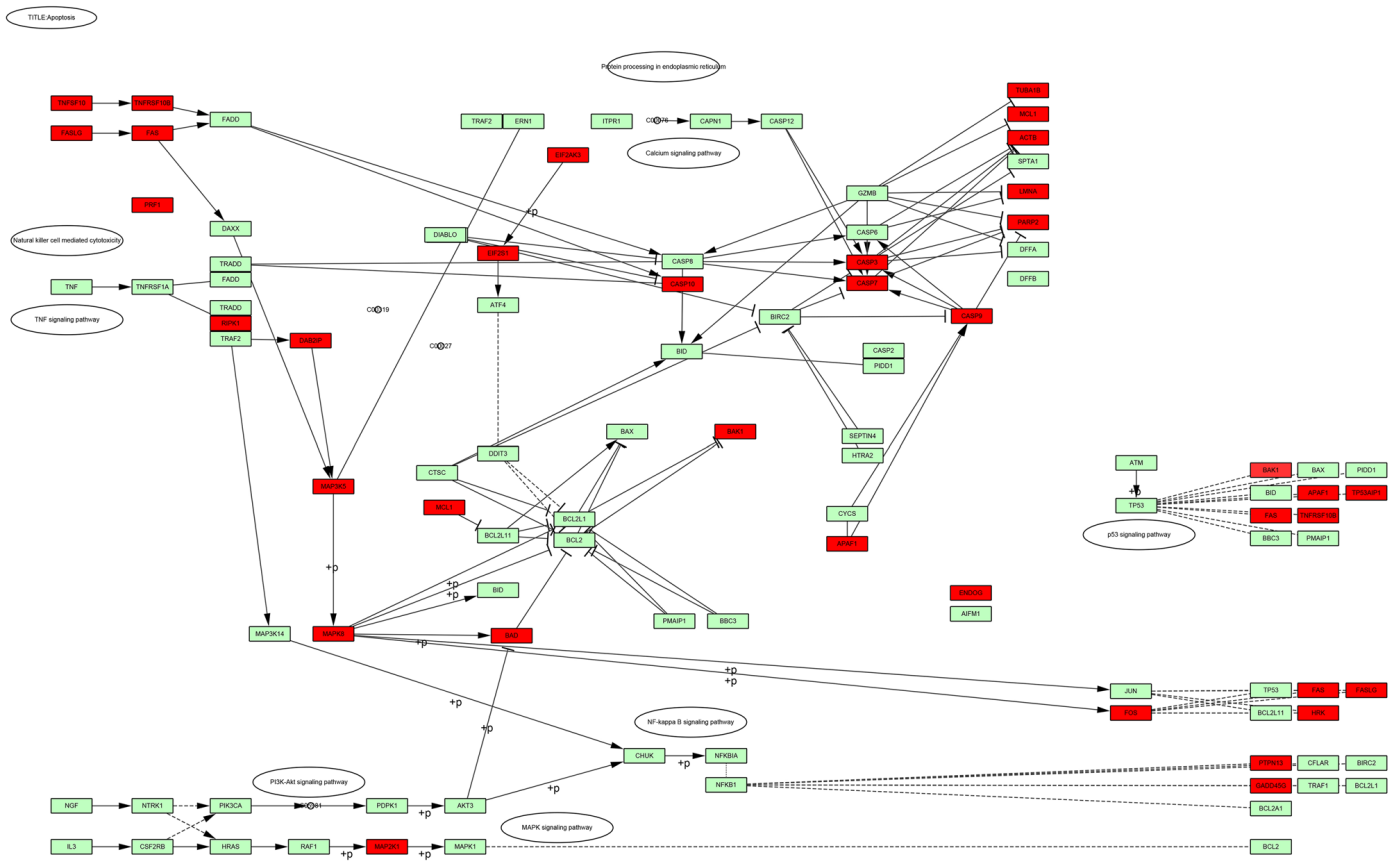
Apoptotic signalling pathways related to *TCN1*. Red represents the pathways correlated with apoptotic pathways.

GO and KEGG analysis was performed, and a total of 5,857 differentially expressed genes (2,683 positively related and 3,174 negatively related, false discovery rate < 0.01) correlated with TCN1 were detected. Spearman’s test was conducted to analyse correlations between *TCN1* and genes differentially expressed in CRC (red represents positively related genes and blue represents negatively related genes) (Fig. 5A). The top 50 genes that were positively and negatively correlated with TCN1 are shown in heat maps (Fig. 5B, C). Over-representation analysis (ORA) was conducted in the top 50 genes positively correlated with *TCN1*. GO analysis showed that the identified *TCN1*-related gene products were mainly expressed in the cell membrane, Golgi apparatus, endoplasmic reticulum, and vesicles, and are involved in cytokine production, protein glycosylation, apoptosis, exocytosis, and vesicle transport (Fig. 6A–C). KEGG pathway analysis showed that cancer-related signalling pathways were enriched, including immunity, apoptosis, platinum resistance, and metabolic, hypoxia-inducible factor-1A (HIF-1A) and vascular endothelial growth factor (VEGF) signalling pathways (Fig. 6D). Molecules (marked in red) connected with TCN1 are presented in an apoptotic pathway network in Fig. 7.

## Discussion

TCN1 is a vitamin B12-binding protein that is present in the cytoplasm and performs functions with TCN2 and intrinsic factors in the processes of cobalamin transport, metabolism, and homeostasis^13,14^. Studies have shown aberrant serum levels of vitamin B12 and TCN1 in some solid tumours^15–19^. Upregulation of TCN1 is associated with tumourigenesis and progress^20–25^. Chu’s meta-analysis of gene expression data based on microarrays demonstrated that *TCN1* act as an oncogene and is overexpressed in CRC^10^. We hypothesized that TCN1 might have a role in colon cancer. The NGS results in CRC revealed that *TCN1* ranked as the second most upregulated mRNA^12^. Bioinformatic analysis based on the COAD data set verified that *TCN1* was indeed overexpressed in CRC. qRT-PCR and western blot assays also verified that TCN1 was highly expressed at both the mRNA and protein level in CRC tissues. All these results provided more evidence that TCN1 has a role in the tumourigenesis of CRC^10,11^, and suggested that TCN1 could be a potential novel biomarker.

IHC in TMAs proved that TCN1 was highly expressed in colon cancer tissues and demonstrated that high expression levels were correlated with advanced pathological features. Pulmonary metastatic tumour tissue from colon cancer also showed high expression of TCN1 and the expression level was in accordance with that in the primary colon cancer tissues. Thus, it could be inferred that TCN1 performed roles in colon cancer progression and metastasis. Lee et al. found that overexpression of TCN1 in rectal cancer was related to advanced pre-/post-treatment tumour status, nodal status, vascular invasion, and tumour regression grade^21^. Wu et al. also reported that TCN1 was highly expressed in pancreatic cancer^26^ and Chu et al. found it had a role in tumour invasion and metastasis, which were all consistent with our findings.

Patients with high expression of TCN1 showed shorter OS and poor prognosis. Previous studies demonstrated that high expression of TCN1 was related to poor prognosis of hypopharyngeal squamous cell and rectal cancer^20,21^. Our findings were concordant with these reports. Univariate analysis proved that TNM stage and TCN1 expression were prognostic factors, while multivariate analysis showed that TCN1 expression and M classification were independent prognostic factors. Further, we compared the expression levels in right- and left-sided colon cancer. Interestingly, TCN1 expression levels were much higher in right-sided than in left-sided colon cancer and the survival time was accordingly shorter, which was paralleled with the literature^27^. This provided further evidence that TCN1 expression was associated with colon cancer behaviour and prognosis.

We found that there was no significant difference in TCN1 expression between colonoscopy biopsy tissues and surgical specimens, which suggested that biopsy tissues obtained by colonoscopy might represent surgical specimens for the detection of TCN1 expression, and thus would make detection easier. Nevertheless, only a small cohort from a single centre was examined, prospective experiments with a larger sample size should performed to confirm the results. We also discovered that the expression level of TCN1 decreased after administration of platinum-containing chemotherapy. It could be inferred that the chemoradiotherapy will affect TCN1 expression through some mechanism. Zhang et al. screened differentially expressed genes from the Gene Expression Omnibus database and revealed that aberrant expression of *TCN1* and *TGFBI* was regulated by DNA methylation^28^. Lee et al. performed in vitro assays and verified that downregulation of *TCN1* led to an obvious reduction in the proliferation of FaDu cells. Knockdown of *TCN1* significantly sensitized FaDu cells to cisplatin treatment, thus suggesting that it might function through an apoptosis pathway^21^. Researchers also proved that *TCN1* expression levels were associated with neoadjuvant chemotherapy sensitivity in hypopharyngeal squamous cell cancer and chemoradiotherapy sensitivity in rectal cancer^20,21^, but the underlying mechanism remains unclear.

To reveal the potential downstream mechanism of *TCN1* in CRC, genes related to *TCN1* were selected to determine the way it functions. Because *TCN1* might have a role as an oncogene^10^, genes positively correlated with *TCN1* were finally chosen for GO and KEGG analysis. As expected, the result proved that *TCN1* is associated with many cancer-related genes and pathways, which provided evidence that *TCN1* might affect tumourigenesis and progress by affecting transcription. Apoptotic pathways were enriched in the KEGG analysis and verified that lysosomes and apoptotic proteins of caspase 3 were located in a prominent location in the pathway network, suggesting that *TCN1* may partially function through the apoptotic pathway. In the meantime, we found that TCN1 was significantly associated with immune response pathways, which provided a new avenue for our future study. Nevertheless, an experiment has not undertaken to verify this inference, which is the limitation of our study. We are focusing on this area and will explore the underlying mechanism in our future work.

To our knowledge, this is the first report to discuss the relationship between expression of *TCN1* and colon cancer behaviour. The research proves that *TCN1* is associated with prognosis and presents clinic evidence for previous research^10,11^. It also provides a new area for future study, especially the underlying mechanism of tumour invasion and the mechanism of predicting chemosensitivity.

Taken together, we conclude that TCN1 expression is significantly overexpressed in colon cancer and is correlated with advanced pathological features. TCN1 may be a predictor of prognosis and a potential biomarker for predicting chemosensitivity. However, further in *vivo* and in *vitro* experiments should be performed to explore the underlying mechanism.

## Methods

### Ethics statement

This study was carried out in accordance with the guidelines of the Fourth Hospital of Hebei Medical University and Hebei Provincial Tumour Hospital. The human samples were provided by volunteer donors who gave their informed consent, and the study was approved by the Ethical Committee of the Fourth Hospital of Hebei Medical University (ID 2017MEC115).

### Tissue collection

All fresh specimens were collected from January 2018 to June 2019 at Hebei Medical University Fourth Affiliated Hospital. Resected tissues of colon cancer and matched adjacent non-tumour colon mucosal tissues (n = 39) were snap-frozen in liquid nitrogen and stored at − 80 °C for qRT-PCR assay and western blotting. None of the patients were treated by radiotherapy or chemotherapy before surgery.

### RNA extraction, quantitative real-time PCR, and next-generation sequencing

Total RNA was extracted from tissues using the Trizol (Life Technologies, Massachusetts, USA) reagent^12,29^. cDNA was synthesized from the total RNA using the GoScript Reverse Transcription System (Promega, Wisconsin, USA) according to the manufacturer's instruction. A SYBR Green PCR kit (Promega, Wisconsin, USA) was used in the amplification process with a 7,500 Real-time PCR system (Applied Biosystems, Massachusetts, USA). The primers (HQP017978, Eockville, MD, USA) were purchased from GeneCopoeia. GAPDH was used as an internal control. The qRT-PCR results were analyzed with the 2^−ΔΔCt^ method. All the reactions were conducted in triplicate and the values are shown as means ± SD. mRNA data were selected from our earlier NGS results^11^, which had been deposited in GEO database (GSE104836, https://www.ncbi.nlm.nih.gov/ geo/query/acc.cgi?acc = GSE104836). The differential expression of the genes was assessed by limma package^30^ (https://bioconductor.org/ packages/release/bioc/html/limma.html) in R Software using RNA-seq read counts. |log2FoldChange|> 2 and adjusted *P* < 0.05 as cut-off criteria for screening the differentially expressed genes (DEGs). A heatmap and volcano plot were drawn using pheatmap and ggplot2 packages in R software to indicate the DEGs.

### Databases verification

The relative mRNA expression level of *TCN1* in a colon adenocarcinoma (COAD) data set were analysed based on an online database: Gene Expression Profiling Interactive Analysis (GEPIA, https://gepia.cancer-pku.cn/)^31^. The database is based on The Cancer Genome Atlas (TCGA) and Genotype-Tissue Expression (GTEx). ANOVA was used for the comparison of tumour tissues with paired normal tissues, with the following thresholds: |log_2_FC|= 1 and *P* = 0.05.

### Western blot analysis

The tumor tissues and their paired adjacent normal colon mucosa were collected. Total proteins of each group were extracted with RIPA buffer containing protein inhibitors (Solarbio, Beijing, China) and quantified by BCA kit (Thermo Fisher Science). The protein cleavage products were separated by 10% sodium dodecyl sulfate–polyacrylamide gel electrophoresis and transferred to polyvinylidene fluoride membrane (Solarbio, Beijing, China). Sealed with 5% skim milk at room temperature for 1 h, and incubated overnight with rabbit anti-human TCN1 monoclonal (a6414 ABclonal, Wuhan, China) at 4 °C. Then washed with Tris buffer containing Tween (TBST) for 3 times, and incubated with HRP labelled goat anti-rabbit second antibody (AS014, ABclonal, Wuhan, China) for 1 h at room temperature. After washing with TBST for 3 times, the signal was detected by chemiluminescence. GAPDH (ab9485, Bioworld, St Louis, MN, USA) was used as a protein loading control. The intensity of protein fragments was quantified with Image J software.

### Tissue microarray (TMA)

TMA slices were obtained from the Department of Pathology, Hebei Medical University Fourth Affiliated Hospital. The TMA, which included 194 cases of colon cancer and paired adjacent normal mucosal tissue, 37 cases of pulmonary metastatic tumours from colon cancer and adjacent normal lung tissues, and 42 cases of colonoscopy biopsy and paired post-chemotherapy colon cancer specimens (these patients received platinum-containing neoadjuvant chemotherapy), was constructed from formalin-fixed, paraffin-embedded tissue blocks. Enrolled patients were followed up by the Department of Follow-up Centre, Hebei Medical University Fourth Affiliated Hospital, and all clinical data were collected. The final follow-up date was 20 May 2019 and the follow-up endpoint was overall survival (range 5.2–76.3 months) with a median follow-up period of 67.5 months.

### Haematoxylin–eosin (HE) staining and immunohistochemistry (IHC)

HE staining was performed for tumour diagnosis, and TCN1 expression was detected using IHC in tumour and adjacent normal tissues. Continuous sections with a thickness of 4 μm were performed for HE and IHC staining on Envision. Anti-TCN1 monoclonal antibody was purchased from AB-clonal (1:60, a6614, ABclonal, Wuhan, China). All slides were interpreted by two pathologists independently who were blinded to the clinical information. The IHC staining score included the proportion of positively stained tumour cells and the staining intensity. The proportion of positively stained tumour cells was scored as follows: 0 (no tumour cells stained), 1 (< 25% tumour cells stained), 2 (25–50% tumour cells stained), 3 (50–75% tumour cells stained), and 4 (75–100% tumour cells stained). The grading of staining intensity was evaluated by the following criteria: 3 (brown, strong staining), 2 (yellow brown, moderate staining), 1(light yellow, weak staining), and 0 (no staining). The final total staining score was calculated by multiplying the proportion of stained tumour cells and the staining intensity score. TCN1 expression was scored and a score of ≤ 3 indicated negative TCN1 expression, while a score of > 3 was considered as positive TCN1 expression. A score of ≤ 6 was designated as low expression and a score of > 6 as high expression. The pathological diagnosis was made in accordance with the histological classification of tumours developed by the World Health Organization.

### GO and KEGG enrichment analysis of *TCN1*-related genes in CRC based on the LinkedOmics database

The LinkedOmics database (https://linkedomics.org), which has been used to analyse TCGA cancer-associated datasets^27^, was selected to identify the differentially expressed genes related to *TCN1*. The database included 379 CRC cohorts. Results from LinkedOmics were assigned and ranked. GO functional enrichment analysis and KEGG pathway analysis were performed by gene set enrichment analysis (GSEA). Spearman’s test was conduct to perform statistical analyses. All the results were graphically showed in heat maps and volcano plots. Linkfinder-related modules were selected to show the results of differentially expressed genes and the network analyses.

### Statistical analysis

Statistical analysis was performed using the software package SPSS 21.0 (Chicago, IL, USA) and figures were constructed with Prism5.0 (GraphPad, Lnc., La Jolla, CA, USA) and R (version: 3.6.1). Correlations between TCN1 expression and clinicopathological features were determined using chi-squared tests or Fisher’s exact tests, as appropriate. The endpoints were defined as patient death or the final follow-up date. Overall survival (OS) was defined as the time between surgery time and death or the last follow-up visit. Kaplan–Meier survival analysis was conducted to plot survival curves and log-rank tests were used to evaluate the prognostic differences between subgroups. Cox multivariate regression analysis was conducted after prognostic significance at the univariate level was determined. A *P *value of < 0.05 was considered statistically significant.

### Ethics approval

We confirm that all the methods had been carried out in accordance with the relevant guidelines and regulations of the Declaration of Helsinki.
